# 973. Pharmacokinetic/Pharmacodynamic Evaluation and Dose Optimization of Daptomycin in Pediatric Patients with *Staphylococcus aureus* Bacteremia

**DOI:** 10.1093/ofid/ofad500.028

**Published:** 2023-11-27

**Authors:** Katie B Olney, Joel I Howard, David Burgess

**Affiliations:** University of Kentucky HealthCare, Lexington, Kentucky; University of Kentucky HealthCare, Lexington, Kentucky; UK HealthCare, Lexington, KY

## Abstract

**Background:**

Optimal dosing of daptomycin (DAP) in pediatric patients has not been elucidated in clinical practice. This study was conducted to examine DAP exposures achieved with package label dosing (PLD) and identify dosing regimens necessary to optimize efficacy and mitigate toxicity in pediatric patients being treated for *Staphylococcus aureus* bacteremia.

**Methods:**

Pharmacokinetic/pharmacodynamic (PK/PD) models were constructed using age-specific pediatric PK parameters from previously published clinical trials in children to determine the probability of target attainment (PTA) for each of the following age cohorts: 3 to 6 months, 7 to 12 months, 13 to 24 months, 2 to 6 years, 7 to 11 years, and 12 to 17 years. Achievement of area under the curve over 24 hours (AUC_0-24_) ≥ 666 mg*hr/L was used as the PD target to determine the PTA for efficacy (PTA_E_). The probability for which the trough concentration (C_min_) exceeded 24.3 mg/L was the PD target used to determine the PTA for toxicity (PTA_T_). These PD targets were selected based on prior adult data correlating DAP exposure with efficacy and safety outcomes. Optimal dosing regimens were considered to be those which achieved the combined target of PTA_E_ ≥ 90% and PTA_T_ ≤ 5%.

**Results:**

If targeting previously validated efficacy (AUC_0-24_ ≥ 666 mg*hr/L) and safety (C_min_ < 24.3 mg*hr/L) endpoints, current pediatric dosing regimens do not achieve adequate DAP exposure for treatment of *Staphylococcus aureus* bacteremia. PLD failed to achieve adequate PTA_E_ in all age groups with only 26.3% PTA_E_ in children 13 to 24 months, 39.5% PTA_E_ in children 2 to 6 years, 30.1% PTA_E_ in children 7 to 11 years, and 50.1% PTA_E_ in children 12 to 17 years of age. Optimal dosing regimens, defined as achieving at least 90% PTA_E_ with PTA_T_ ≤ 5%, are displayed in **Table 1**.

**Figure:**
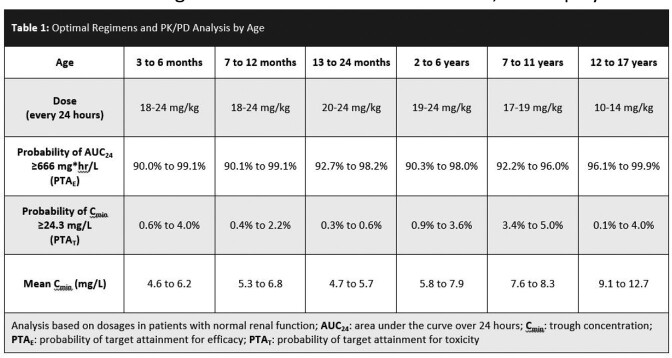

**Conclusion:**

Current PLD of DAP resulted in inadequate exposure for all pediatric patients evaluated. If using validated PD targets for efficacy and safety, adequate PTA_E_ and minimal PTA_T_ were achieved with DAP at dosages of 20 mg/kg every 24 hours in children 3 months to 6 years of age, 18 mg/kg every 24 hours in children 7 to 11 years of age, and 10 mg/kg every 24 hours in children 12 to 17 years of age. Clinical studies evaluating these augmented dosages need to be conducted.

**Disclosures:**

**Katie B. Olney, PharmD, BCIDP**, The Society of Infectious Diseases Pharmacists (SIDP): Grant/Research Support

